# Potato Consumption and Risk of Type 2 Diabetes Mellitus: A Harmonized Analysis of 7 Prospective Cohorts

**DOI:** 10.1016/j.tjnut.2024.07.020

**Published:** 2024-09-16

**Authors:** Luc Djousse, Xia Zhou, Jaewon Lim, Eunjung Kim, Howard D Sesso, I-Min Lee, Julie E Buring, Robyn L McClelland, John M Gaziano, Lyn M Steffen, JoAnn E Manson

**Affiliations:** 1 Department of Medicine, Brigham and Women’s Hospital, Boston, MA, United States; 2 Department of Medicine, Harvard Medical School, Boston, MA, United States; 3 Division of Epidemiology and Community Health, School of Public Health, University of Minnesota, Minneapolis, MN, United States; 4 Department of Biostatistics, University of Washington, Seattle, WA, United States

**Keywords:** epidemiology, risk factor, type 2 diabetes, diet, potato consumption

## Abstract

**Background:**

Data on the relation of potato consumption with risk of type 2 diabetes (T2D) are limited and inconsistent. It is unclear whether the plant-based diet index (PDI), which is a novel and comprehensive tool to assess overall dietary pattern, modifies the association of potato intake with T2D.

**Objectives:**

We examined the association of total, combined baked, boiled, and mashed potatoes and fried potatoes with risk of T2D and test the interaction between PDI score and potato consumption on T2D risk.

**Methods:**

We conducted a de novo, harmonized, individual-level data from 7 United States cohorts (*N* = 105,531). Cox regression was used to estimate hazard ratios (HRs) separately in each cohort adjusting for anthropometric, demographic, and lifestyle factors and cohort-specific results were pooled using an inverse-variance weighted method.

**Results:**

Mean age ranged from 25 to 72 y, 65% women, and mean consumption of total potatoes ranged from 1.9 to 4.3 times per week. In the primary analysis, total potato intake was not associated with T2D risk: multivariable adjusted HR of 1.01 (95% confidence interval [CI]: 0.95, 1.08) for consumption of 1–2 servings/wk; 1.01 (95% CI: 0.93, 1.10) for >2–3 servings/wk; 1.05 (95% CI: 0.99, 1.12) for >3 to <5 servings/wk; and 1.07 (95% CI: 0.99, 1.16) for 5+ servings/wk compared with no potato intake. In secondary analyses, consumption of combined baked, boiled, and mashed potatoes was not associated with T2D risk, whereas fried potato consumption was positively associated with T2D risk: HR were 1 (ref), 1.07 (95% CI: 1.02, 1.12), and 1.12 (95% CI: 1.03, 1.22) for intake frequency of 0/wk, >0 to 1/wk, and >1/wk, respectively (*P*-trend = 0.04). There was no significant interaction between PDI score and potato consumption on T2D risk.

**Conclusions:**

Although consumption of total potato is not associated with T2D risk, a modest elevated risk of T2D is observed with fried potato consumption.

## Introduction

Consumption of potatoes is highly prevalent in the United States and worldwide [1]. Potatoes are rich in starch; vitamin C; potassium that is known to lower blood pressure [2,3] and stroke risk [3,4]; iron; and carbohydrates, which can influence glucose metabolism, especially postprandial glycemia and insulin secretion [5]. Potatoes have a relatively high glycemic index and load—2 factors that have been shown in some studies to increase risk of type 2 diabetes (T2D) [6,7]. Current data on the association of total potato consumption and risk of T2D have been limited and inconsistent. Although a prospective cohort of Chinese women reported an inverse association of total potato consumption with T2D [multivariable adjusted relative risk (RR) for T2D of 1.00 (ref), 0.82, 0.69, 0.78, and 0.72 across consecutive quintiles of total potato intake] [8], a meta-analysis of 7 studies found a positive association of total potato consumption with T2D risk: every 150 g/d increase of total potatoes was associated with 18% higher risk of T2D (hazard ratio [HR]: 1.18; 95% confidence interval [CI]: 1.10, 1.27; with modest heterogeneity of *I*^2^ = 30%), with a stronger association for French fries (RR: 1.66; 95% CI: 1.43, 1.94; per 150-g/d increase in French fries) than for a combination of baked, boiled, and mashed potato intake (corresponding RR: 1.09; 95% CI: 1.01, 1.18) [9]. Few other studies have evaluated the relation of types of potato preparation (i.e., baked, boiled, mashed, or fried potatoes) with T2D risk. A meta-analysis of 3 cohorts showed 4% higher risk of T2D for every 3 servings/wk of baked, boiled, or mashed potatoes (RR: 1.04; 95% CI: 1.01, 1.08) and 19% higher risk of T2D for French fries (HR: 1.19; 95% CI: 1.13, 1.25) [10].

The inconsistency of results on potato consumption and T2D risk could be partially explained by inadequate control for overall dietary patterns [i.e., plant-based dietary index (PDI), healthy plant-based dietary index (hPDI), and unhealthy plant-based dietary index (uPDI)] [11], residual confounding by other dietary factors, limited statistical power, range of intakes, and/or a lack of consideration of method of preparation. It is common in the United States to consume French fries with red/processed meat, which has been associated with higher risk of T2D [12]. Hence, inadequate control of foods consumed with potatoes may confound the association between potato intake and T2D. We chose the PDI as a tool to account for dietary pattern over other dietary patterns (i.e., Dietary Approach to Stop Hypertension or Mediterranean Diet) because PDI score not only is based on most items included in Dietary Approach to Stop Hypertension (fruits, vegetables, whole grains, nuts/legumes, sugar-sweetened beverages, dairy, and red meat) or Mediterranean Diet (fruits, vegetables, nuts, legumes, whole grains, fish, and red meat) but also includes additional food items such as tea/coffee, refined grains, fruit juice, sweets/desserts, animal fat, eggs, and miscellaneous (such as pizza, chowder, and cream soup). Using data from 7 large United States cohorts, this project examined primarily the association of total potato consumption with T2D risk and secondarily relations of combined baked, boiled, and mashed as well as fried potatoes with T2D risk. In tertiary analyses, we evaluated potential interaction between overall dietary patterns (as measured by PDI, hPDI, and uPDI) and potato consumption on T2D risk.

## Methods

### Study population

We used data collected on 7 prospective cohorts that agreed to participate in the grant application and had data on both exposure and outcomes of interest (Supplemental Table 1). We excluded subjects with prevalent diabetes and/or missing data on diet. Details on exclusion criteria within each cohort are provided in Supplemental Flow Charts a–g.

#### Women’s Antioxidant Cardiovascular Study

The Women’s Antioxidant Cardiovascular Study (WACS) was a randomized, double-blind, placebo-controlled, factorial trial of vitamin C, vitamin E, β-carotene, and folic acid/vitamin B-6/vitamin B-12 in the prevention of cardiovascular events among 8711 women aged 40+ y with a history of cardiovascular disease (CVD) or high CVD risk. A detailed description of WACS has been published [13].

#### Women’s Health Study

The Women’s Health Study (WHS) was a randomized trial designed to assess the effects of aspirin and vitamin E on CVD and cancer among 39,876 healthy female professionals aged 45+ y. Upon completion of the trial in 2004, 33,682 of the original participants who consented agreed to be followed through annual questionnaires for occurrence of cardiometabolic events including T2D and other outcomes. A detailed description has been published previously [14].

#### The Physicians’ Health Study

The Physicians’ Health Study (PHS) I was a randomized, double-blind, placebo-controlled trial designed to study low-dose aspirin and β-carotene for the primary prevention of CVD and cancer among 22,071 United States male physicians between 1982 and 1995. The PHS II was a completed randomized trial designed to assess the effects of vitamin supplements on CVD and cancer between 1997 and 2011. PHS II enrolled 7641 members of the PHS I as well as 7000 newly recruited male physicians. Participants of PHS I and II have been followed up annually using questionnaires. Detailed description of PHS I and II can be found in previous publications [15,16].

#### The Multi-Ethnic Study of Atherosclerosis

The Multi-Ethnic Study of Atherosclerosis (MESA) is a multi-ethnic cohort study of 6814 adults aged 45–84 y and free of clinical CVD at baseline designed to investigate the prevalence and progression of subclinical CVD. A detailed description of MESA has been published [17].

#### The Atherosclerosis Risk in Communities study

The Atherosclerosis Risk in Communities (ARIC) study is a community-based prospective cohort study of 15,792 participants aged 45–64 y at baseline and recruited from 4 communities in the United States starting in 1987. The goal of the ARIC study is to investigate the etiology of atherosclerosis and its consequences and variation in cardiovascular disease risk factors, medical care, and disease by race, sex, place, and time. Details on the design and methods of the ARIC study have been published [18].

#### The Coronary Artery Risk Development in Young Adults study

The Coronary Artery Risk Development in Young Adults (CARDIA) study is a multicenter longitudinal study to elucidate the etiology of coronary artery disease in 5115 Black and White adult men and women who were aged 18–30 y at baseline (1985–1986) and free of CVD. A detailed description of the CARDIA study has been published [19].

#### The Cocoa Supplement and Multivitamin Outcomes Study.

The Cocoa Supplement and Multivitamin Outcomes Study (COSMOS) was a randomized, double-blind, placebo-controlled, 2 × 2 factorial trial testing the efficacy of multivitamin supplements and cocoa extract supplements on cancer and CVD among 21,442 United States adults from 2016 to 2020. Details on the design and methods of COSMOS study have been published [20,21].

Participants provided informed consent and each study was approved by the respective institutional review boards. This project was approved by the institutional review board at Mass General Brigham (Protocol # 2022P001795, PI: LD).

### Assessment of potato consumption

Consumption of potatoes was assessed in each cohort using food questionnaires (Supplemental Table 1). The Willett food frequency questionnaire has been validated previously [22,23]. The potato question specified the portion size, and each participant was asked to report the frequency of intake of various forms of potatoes over the previous 12 mo as described further.

#### ARICs

Participants reported consumption of French-fried potatoes (4 oz); baked (1) or mashed potatoes (1 cup) potatoes; and potato chips or corn chips (a small bag or 1 oz) during visit 1. Response categories were almost never, 1–3/mo, 1/wk, 2–4/wk, 5–6/wk, 1/d, 2–3/d, 4–6/d, and >6/d.

#### Coronary Artery Risk Development in Young Adults

In CARDIA, subjects reported frequency of consumption of fried potatoes including French fries (1 serving = 70 g); hash browns, pan fried potatoes, and potato tots (1 serving = half a cup); boiled (1 serving = half a cup); baked (1 serving = 1 medium); and other potatoes (1 serving = half a cup).

#### Multi-Ethnic Study of Atherosclerosis

Participants reported their consumption of French fries, fried potatoes, hash brown; boiled/baked/mashed or other potatoes, turnips; and potato chips and corn/tortilla chips. Subjects were provided with 3 portion sizes to choose from (small, medium, large). Prespecified answers were rare/never, 1/mo, 2–3/mo, 1/wk, 2/wk, 3–4/wk, 5–6/wk, 1/d, and 2+/d.

#### COSMOS, PHS, WACS, and WHS

For these cohorts, subjects were asked to report the frequency of consumption of French fries (serving size of 4 oz except COSMOS with 6 oz); baked/boiled (1) or mashed (1 cup) potatoes; and potato chips or corn chips (small bag or 1 oz). Response categories were never/<1/mo, 1–3/mo, 1/wk, 2–4/wk, 5–6/wk, 1/d, 2–3/d, 4–5/d, and 6+/d.

We converted reported frequencies into servings per week using the midpoint for interval responses. For potato consumption with open upper boundary, we multiplied the lower boundary by 1.5 to obtain an estimate of average potato consumption in that category as previously described for open ended categories [24]. For each subject, we summed the frequencies for fried, baked, boiled, and mashed potatoes to obtain total potato consumption. We kept the combined baked, boiled, and mashed potatoes as a single group. We did not include potato chips because most cohorts did not separate potato chips from corn or tortilla chips.

### Assessment of T2D

In MESA, T2D was defined as a fasting glucose of ≥126 mg/dL and/or reported use of diabetes medications during in-person clinic examinations at any point during follow-up [25]. In ARIC study, T2D was defined by a self-reported physician diagnosis, self-reported use of diabetes medications, a nonfasting blood glucose concentration of ≥200 mg/dL, or a fasting blood glucose of ≥126 mg/dL [26]. In CARDIA study, T2D was defined as fasting glucose of ≥7.0 mmol/L or the use of diabetes medications during follow-up [27]. In COSMOS, PHS, WACS, and WHS, T2D was ascertained via self-report of T2D diagnosis and/or use of diabetes medications [28,29].

### Definition of PDI, hPDI, and uPDI

To compute overall PDI as well as hPDI and uPDI, we grouped food items as follows: 6 healthy plant food groups (whole grains, fruits, vegetables, nuts, legumes, and tea/coffee); 4 unhealthy plant food groups excluding potatoes (fruit juices, sugar-sweetened beverages, refined grains, and sweets/desserts); and 6 animal food groups [animal fats, dairy, eggs, fish/seafood, meat (poultry and red/processed meat), and miscellaneous animal-based foods (i.e., pizza, chowder, or cream soup)]. We then created quintiles for each of the 16 foods and assigned a score from 1 to 5 from the lowest to the highest.

For the overall PDI, higher intakes of healthy and unhealthy plant food groups received higher scores (score of 1 to 5 from the lowest to the highest quintile), whereas reverse scoring were used for animal food groups (e.g., score of 5 for first quintile of butter and score of 1 for the fifth quintile of butter) as described previously [30]. For the hPDI, healthy plant food groups received higher scores whereas unhealthy plant food groups and animal food groups were assigned reverse scores. For the uPDI, unhealthy plant food groups received positive scores, whereas healthy plant food groups and animal food groups received reverse scores.

We summed the scores of each of the 17 food groups to obtain PDI, hPDI, and uPDI scores (range: 17–85). A higher score on the PDI reflects a higher intake of plant foods and/or a lower intake of animal foods whereas a higher score on the hPDI indicates a higher intake of healthy plant foods and/or a lower intake of unhealthy plant foods and animal foods and a higher score on the uPDI reflects a higher intake of unhealthy plant foods and/or a lower intake of healthy plant foods and animal foods.

### Other variables

Each cohort has collected baseline information on age, sex, race/ethnicity, education, smoking, alcohol intake, physical activity, dietary intake, and BMI. Dietary intake includes fruits and vegetables, sugar-sweetened beverages, legumes, nuts, whole grains, sodium intake, low-fat dairy products, fish intake, fried foods, fresh red meat, processed meat (e.g., bacon, sausage, salami, hot dog), and nutrients including energy intake, transfat, saturated fat, and ratio of polyunsaturated to saturated fats.

### Statistical analysis

Cohort-specific analyses were completed by local investigators using a standardized and uniform data analysis plan and cohort-specific results were meta-analyzed at Brigham and Women’s Hospital. Covariate harmonization occurred at the beginning of data analysis where investigators from all cohorts made a priori decision on whether covariates will be continuous (i.e., age, BMI, food items, MET-h/week) or categorical using cohort-specific categories (i.e., alcohol consumption, physical activity in the absence of MET-h/wk) or quintiles. We excluded subjects with missing data on potatoes and prevalent T2D (Supplemental Flow Charts a–g). We initially created fixed categories of various potato consumption and merged adjacent categories in the presence of sparse events. For the primary analysis, total potatoes categories were <1, 1–2, >2 to 3, >3 to <5, and 5+ servings/wk. Corresponding categories for secondary analyses were <0.5, 0.5–1, >1 to 3, and >3 servings/wk for combined baked, boiled, and mashed potatoes and 0, >0 to 1, and >1 servings/wk for fried potatoes. Person-time of follow up was computed from the time of potato assessment until the first occurrence of T2D, death, or end of the respective study follow-up. Incidence of T2D was calculated by dividing the number of new T2D cases by the person-time of follow up in the respective potato category. Cox proportional hazard models were fitted to estimate HRs with 95% CIs. Proportionality of hazards over time were tested using Schoenfeld residuals and were met. After the crude model, we built sequential models based on a priori knowledge on confounding factors and potential mediators. Model 1 controlled for age, sex, race, education, energy intake, smoking status, alcohol intake, and physical activity; model 2 adjusted for model 1 plus PDI score; model 3 adjusted for model 1 plus fruits and vegetables, red/processed meat, whole grain, sugar-sweetened beverages, nuts/peanut butter, legumes, and transfatty acids (when available); and model 4 adjusted for model 3 plus BMI. We assessed confounding by using change in HRs obtained from model 1 and subsequent models [[2], [3], [4]] and because model 3 showed the maximum change in HR, we retained model 3 for current results including figures. To calculate the *P* value for linear trend, we created a new variable that assigned midpoint of the potato category and fit it as a continuous variable in a regression model. In tertiary analyses, we evaluated the interaction between PDI, hPDI, and uPDI by including the product term of these variables with potato variable in a hierarchical multivariable Cox regression model. We also conducted stratified analyses by BMI (<25 compared with 25+ kg/m^2^).

HRs obtained from cohort-specific analyses were used for fixed-effect meta-analyses using inverse weighted variance [31]. We assessed heterogeneity using Q statistic, I^2^, and visualized using Galbraith plot in STATA SE, version 15. We evaluated the presence of influential study using removal of one study at a time method. To assess dose-response relation and evaluate the shape of the potato–T2D association, we used generalized least squares regression described by Greenland and Longnecker [32] and fit cubic splines with knots at 0.75, 2.0, and 3.5 servings/wk of total potato consumption. Two-sided *P* value was used with α level of 0.05.

## Results

Of 105,531 subjects included in this analyses, 64.8% were women. The mean age at baseline ranged from 25.1 (CARDIA) to 72.0 y (COSMOS) and mean total potato consumption ranged from 1.9 (MESA) to 4.2 (CARDIA) times/wk. Average consumption of combined baked, boiled, and mashed potatoes ranged from 1.22 (MESA) to 1.50 (WACS) times/wk and corresponding ranges for fried potatoes were 0.43 (WHS) to 1.89 (CARDIA). Baseline characteristics are summarized in Table 1.

**TABLE 1:** Characteristics of 7 cohorts included in current analyses of potato–type 2 diabetes association^1^

Characteristics	Cohorts						
	ARIC (*n* = 15,106)	CARDIA (*n* = 4042)	COSMOS (*n* = 16,808)	MESA (*n* = 5,417)	PHS (*n* = 19,472)	WACS (*n* = 6500)	WHS (*n* = 38,186)
Age (y)	54.2 ± 5.8	25.1 ± 3.5	72.1 ± 6.6	62.4 ± 10.3	66.4 ± 9.3	60.8 ± 8.9	54.6 ± 7.0
Sex (% women)	55.4	57.2	61.0	52.9	0	100	100
White race (%)	73.0	53.6	90.0	61.6	92.6	94.7	95.1
African Americans (%)	26.6	46.4	4.07	26.4	0.75	2.72	2.13
BMI (kg/m^2^)	27.7 ± 5.4	24.5 ± 5.0	27.1 ± 5.0	28.2 ± 5.4	25.7 ± 3.3	29.6 ± 6.4	25.9 ± 5.0
Energy intake (kcal)	1614 ± 566	2384 ± 859	1612 ± 618	1607 ± 716	1686 ± 521	1729 ± 553	1726 ± 534
PDI score	47.9 ± 5.6	47.1 ± 5.4	44.9 ± 6.0	48.0 ± 5.9	48.3 ± 6.4	48.9 ± 6.0	48.4 ± 6.0
hPDI score	47.7 ± 5.9	49.6 ± 7.2	44.9 ± 6.5	48.0 ± 7.2	47.8 ± 6.5	47.8 ± 6.5	48.0 ± 6.5
uPDI score	48.2 ± 6.0	48.7 ± 6.7	44.7 ± 7.5	48.0 ± 6.8	48.3 ± 6.8	48.7 ± 7.0	48.7 ± 6.8
Total potatoes (servings/wk)	2.84 ± 2.34	4.30 ± 4.01	2.16 ± 2.36	1.85 ± 1.84	2.50 ± 2.19	2.96 ± 2.49	2.72 ± 2.17
Baked/mashed/boiled potatoes (servings/wk)	2.11 ± 1.97	2.41 ± 2.99	1.72 ± 2.08	1.22 ± 1.34	1.94 ± 1.82	2.50 ± 2.22	2.29 ± 1.96
Fried potatoes (servings/wk)	0.73 ± 1.08	1.89 ± 2.46	0.45 ± 0.75	0.62 ± 1.03	0.56 ± 0.92	0.46 ± 0.86	0.43 ± 0.71
Alcohol intake (drinks/wk)	3.0 ± 6.6	1.78 ± 1.97	NA	4.01 ± 8.41	NA	NA	NA
Physical activity (MET-h/wk or score^2^)	2.43 ± 0.79	400.5 ± 284.9	25.2 ± 25.3	94.7 ± 97.5	NA	NA	14.6 ± 18.3
Fruit and vegetables (servings/wk)	32.3 ± 17.9	36.4 ± 24.7	33.0 ± 24.3	30.7 ± 18.3	23.5 ± 14.2	36.6 ± 22.1	35.3 ± 21.7
Whole grain intake (servings/wk)	8.48 ± 8.25	10.44 ± 10.22	6.53 ± 6.36	5.46 ± 4.99	11.9 ± 11.8	9.39 ± 8.39	10.4 ± 8.9
Sodium intake (g/d)	1.47 ± 0.57	3.56 ± 1.44	1.35 ± 0.59	2.28 ± 1.14	1.48 ± 0.55	1.78 ± 0.66	1.86 ± 0.66
Red/processed meats (servings/wk)	7.30 ± 5.06	30.1 ± 22.3	7.74 ± 7.73	5.47 ± 4.17	7.48 ± 6.88	8.39 ± 7.52	7.62 ± 6.51
Sugar-sweetened beverages (servings/wk)	3.63 ± 6.67	11.7 ± 13.2	1.24 ± 3.52	2.75 ± 6.04	2.07 ± 3.91	2.70 ± 5.80	22.2 ± 12.2
Fish intake (servings/wk)	2.02 ± 2.08	6.67 ± 9.58	2.14 ± 2.72	2.02 ± 2.27	1.60 ± 1.43	1.87 ± 2.20	1.86 ± 1.83
Legumes (servings/wk)	0.78 ± 1.17	1.60 ± 3.56	3.27 ± 4.00	2.78 ± 4.26	3.66 ± 3.27	3.32 ± 3.01	3.05 ± 2.74
Nuts/peanut butter (servings/wk)	0.88 ± 1.85	3.57 ± 6.81	5.13 ± 6.81	1.82 ± 2.76	1.84 ± 2.78	1.24 ± 2.14	1.32 ± 2.15
Saturated fat intake (g/d)	21.7 ± 10.0	37.4 ± 16.4	20.9 ± 10.08	18.5 ± 11.2	18.4 ± 8.2	19.7 ± 8.6	19.7 ± 8.1
Trans fat intake (g/d)	2.88 ± 1.73	NA	0.85 ± 0.28	3.41 ± 2.28	1.71 ± 0.68	NA	2.28 ± 1.06

Abbreviations: ARIC, Atherosclerosis Risk in Communities; CARDIA, The Coronary Artery Risk Development in Young Adults Study; COSMOS, Cocoa Supplement and Multivitamin Outcomes Study; hPDI, healthy plant-based diet index; MESA, Multi-Ethnic Study of Atherosclerosis; MET-h, metabolic equivalent per hour; NA, not available; PDI, plant-based diet index; PHS, Physicians’ Health Study; T2D, Type 2 diabetes; uPDI, unhealthy plant-based diet index; WACS, The Women’s Antioxidant Cardiovascular Study; WHS, Women’s Health Study.

1 Values are mean ± SD unless specified.

2 Physical activity score for CARDIA and MESA.

**FIGURE 1:**
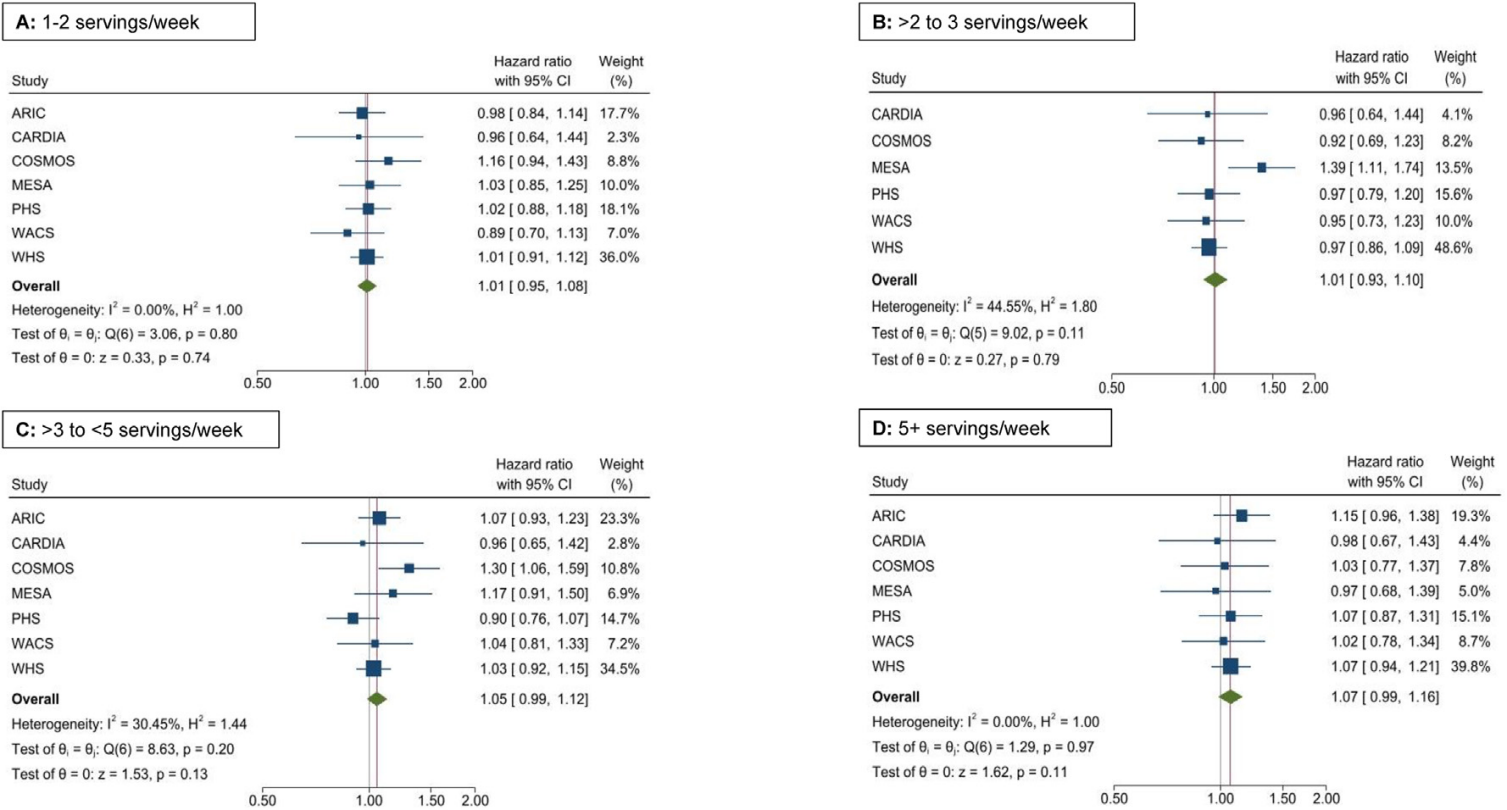
Forest plot depicting association of total potato intake with incidence of T2D in 7 cohorts. Cox regression adjusting for age, sex, BMI, race, education, energy intake, smoking status, alcohol intake, physical activity, fruits and vegetables, red/processed meat, whole grain, sugar-sweetened beverages, nuts/peanut butter, legumes, and trans fatty acids (when available).

**FIGURE 2:**
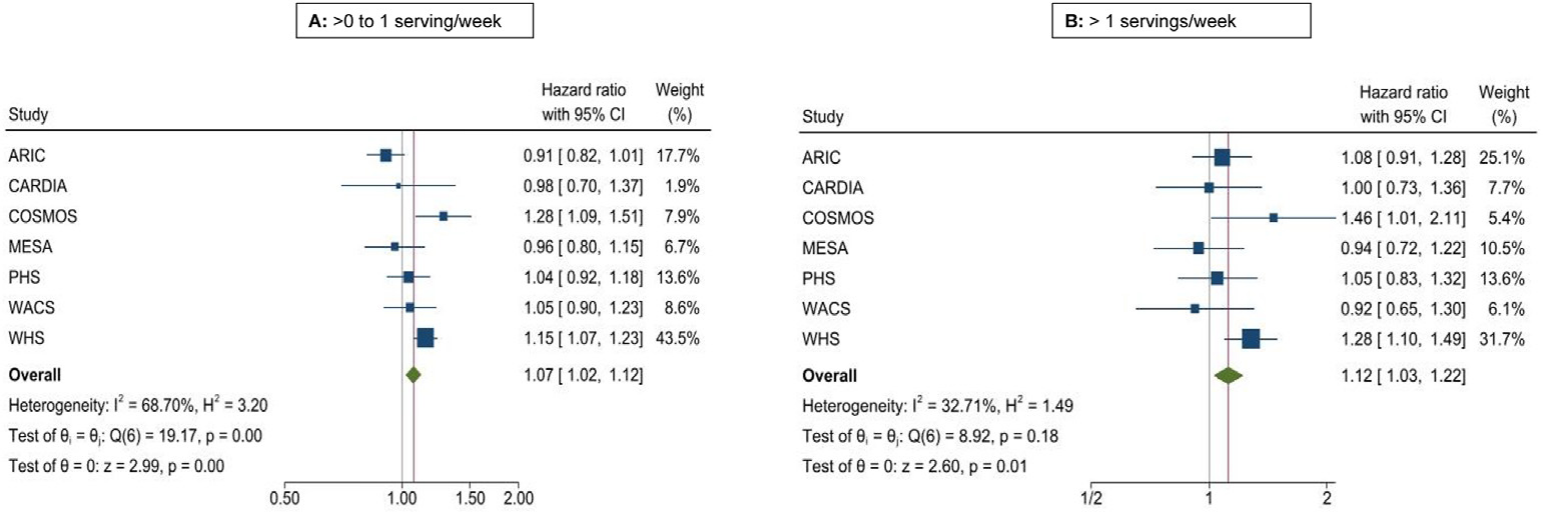
Forest plot depicting association of fried potato intake with T2D risk in 7 cohorts. Cox regression adjusting for age, sex, BMI, race, education, energy intake, smoking status, alcohol intake, physical activity, fruits and vegetables, red/processed meat, whole grain, sugar-sweetened beverages, nuts/peanut butter, legumes, and trans fatty acids (when available).

In the primary analysis, total potato consumption was not associated with risk of T2D with HRs of 1.01 (95% CI: 0.95, 1.08) for total potato consumption of 1–2 servings/wk; 1.01 (95% CI: 0.93, 1.10) for >2 to <3 servings/wk; 1.05 (95% CI: 0.99, 1.12) for 3 to <5 servings/wk; and 1.07 (95% CI: 0.99, 1.16) for 5+ servings/wk compared with no potato intake in a model adjusting for age, sex, race, BMI, energy intake, education, physical activity, smoking, alcohol intake, fruits and vegetables, red/processed meat, whole grain, sugar-sweetened beverages, nuts/peanut butter, legumes, and transfatty acids (when available) (Figure 1). In secondary analyses, consumption of combined baked, boiled, and mashed potatoes was not associated with T2D with multivariable adjusted HR of 1 (ref), 1.00 (95% CI: 0.95, 1.07), 1.01 (95% CI: 0.95, 1.08), and 1.03 (95% CI: 0.95, 1.11) for intake frequency of <0.5/wk, 0.5–1/wk, >1 to 3/wk, and >3/wk, respectively (Supplemental Figure 1). Furthermore, fried potato consumption was positively associated with T2D risk: compared with subjects who did not consume fried potatoes, HR were 1.07 (95% CI: 1.02, 1.12) for fried potato intake of >0 to 1/wk and 1.12 (95% CI: 1.03, 1.22) for fried potato intake of >1/wk (*P*-trend = 0.04) (Figure 2) after adjustment for age, sex, race, BMI, education, energy intake, smoking, alcohol intake, physical activity, fruits and vegetables, red/processed meat, whole grain, sugar-sweetened beverages, nuts/peanut butter, legumes, and transfatty acids (when available). There was a modest heterogeneity in the data (*I*^2^ = 33%–69%) and omitting 1 study at a time showed the greatest attenuation of HR with omission of WHS **(**Supplemental Figure 2).

**FIGURE 3:**
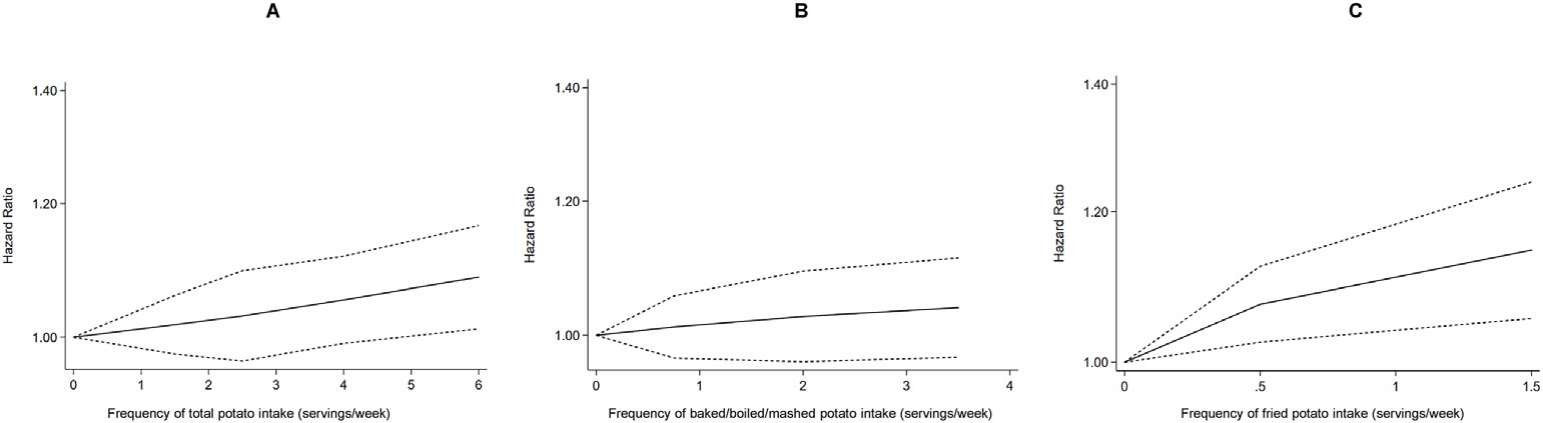
Dose-response relation of total (A), baked/boiled/mashed (B), and fried (C) potatoes with risk of T2D. Restricted cubic spline fitted with multivariable adjusted model controlling for age, sex, BMI, race, education, energy intake, smoking status, alcohol intake, physical activity, fruits and vegetables, red/processed meat, whole grain, sugar-sweetened beverages, nuts/peanut butter, legumes, and trans fatty acids (when available).

In a dose-response analysis using restricted cubic splines, total potato consumption was not associated with T2D risk, and similar result was obtained for combined baked, boiled, and mashed potatoes (Figure 3). In contrast, fried potato intake was positively associated with T2D (Figure 3).

In tertiary analyses, no interaction was seen between PDI, hPDI, or uPDI and potato consumption on T2D risk (all *P*-interaction > 0.05, data not shown). Finally, association of fried potato consumption with T2D was statistically significant only in subjects with BMI ≥ 25 but not <25 kg/m^2^, albeit nonstatistically significant interaction (BMI × fried potatoes) (Supplemental Figure 3).

## Discussion

In this meta-analysis of 7 large United States cohorts, total potato consumption was not associated with higher risk of T2D after adjustment for potential confounding factors. In secondary and tertiary analyses, greater intake of fried potatoes was significantly related to higher T2D risk while combined intake of baked, boiled, or mashed potatoes was not associated with T2D risk and there was no evidence for an interaction between PDI score and potato consumption on T2D risk. A few studies have focused on the method of preparation and no previous study has evaluated the interaction with PDI when assessing the association of potato consumption with incidence of T2D.

### Total potato consumption and risk of T2D

Our findings of no association of total potato consumption with T2D risk are consistent with results of the Framingham Offspring study (*n* = 2523), which reported no association between total potato intake and incidence of T2D or impaired fasting glucose (IFG) (HR: 0.97; 95% CI: 0.81, 1.15) comparing intake of 4+ with <1 cup-equivalent/wk of total potatoes [33]. A study of 64,227 Chinese women without baseline chronic disease had an inverse association of potato intake with incidence of T2D; it is noteworthy that the adult Chinese women were relatively younger (mean age: 50 y) and had a lower prevalence of T2D risk factors including current smoking (2%), physical inactivity (15%), and obesity (5%) [8]. In contrast, the Alpha Omega cohort [34] showed 54% higher risk of T2D comparing the highest to the lowest tertile of total potatoes (95% CI: 4%, 130%). Furthermore, analysis of data from 174,665 subjects of the UK Biobank showed 28% higher risk of T2D (95% CI: 13%, 45%) comparing subjects consuming 2+ servings/wk of total potatoes with those reporting no potato intake [35]. In a meta-analysis of 6 studies, each 100-g/d increase in total potato consumption was associated with 8% higher risk of T2D (HR: 1.08; 95% CI: 1.02, 1.15) with evidence of heterogeneity of *I*^2^ = 55.4 (*P*-heterogeneity = 0.03) [36]. Another meta-analysis suggested heterogeneity by geographic location, with a positive association between total potato intake and T2D risk observed in studies conducted in Western countries (HR: 1.19; 95% CI: 1.06, 1.34; *P*-heterogeneity = 0.09; *n* = 8 studies) but not in Eastern countries (HR: 0.94; 95% CI: 0.71, 1.25; *P*-heterogeneity < 0.0001; *n* = 5 studies) [37]. A recent meta-analysis of 383,211 participants (23,189 T2DM cases) reported 5% higher risk of T2D per 100 g/d increment of total potatoes (HR: 1.05; 95% CI: 1.02–1.08) [38]. It is possible that the heterogeneity reported in the literature on total potato consumption and T2D risk could be partially accounted for by differential relations between baked, boiled, mashed, or fried potatoes and T2D risk and differential consumption of these types of potatoes across cohorts. Therefore, it is important to evaluate the influence of preparation methods of potatoes when assessing risk of T2D.

### Baked, boiled, and mashed potato consumption and risk of T2D

In our meta-analysis, we did not find evidence of an association between combined baked, boiled, and mashed potato intake and the incidence of T2D after adjustment for demographic, anthropometric, and lifestyle factors. These results are consistent with data from the Framingham Offspring study, which reported no association between intake of nonfried potatoes (potatoes that were baked, boiled, or used as ingredients in nonfried dishes) and risk of T2D/IFG [33]. Noticeable differences between our meta-analysis and the Framingham study include the use of combined T2D and IFG as outcome in the Framingham study and the older age of our analysis (mean age >60 y in most cohorts included in our analysis compared with 50 y in Framingham). Contrary to our null findings, the UK biobank reported 69% (HR: 1.69; 95% CI: 1.06, 2.69) higher risk of T2D comparing 2+ serving/wk of mashed potatoes with none whereas intake of baked/boiled potatoes was not associated with T2D risk in the UK biobank [35]. Unfortunately, we did not have data on mashed potato consumption as a separate category to compare with UK biobank findings [35]. A meta-analysis of 4 studies reported a positive association of combined baked, boiled, and mashed potatoes with T2D risk (HR: 1.08; 95% CI: 1.00, 1.16) [37]. In the Alpha Omega cohort [34], boiled potato consumption was positively associated with T2D risk [adjusted HR (95% CI): 1.0 (ref), 1.39 (0.95, 2.05), and 1.60 (1.06, 2.40) across consecutive tertiles of exposure].

### Consumption of fried potatoes and risk of T2D

Our meta-analysis showed slightly elevated risk of T2D with fried potato consumption, and such findings are in line with the data from the Nurses’ Health Study [10], the Alpha Omega cohort [34], and the UK biobank study [35]. A recent meta-analysis reported 10% higher risk of T2D per 100-g/d increment of French fries’ intake (HR: 1.10; 95% CI: 1.07, 1.14) [38]. Contrary to our findings, there was no association between fried potato and incidence of T2D/IFG in the Framingham Offspring study [33]. It is unclear if similar findings would have been observed in Framingham after exclusion of IFG as outcome.

### Biologic mechanisms by which potatoes might influence risk of T2D

Potatoes are high in starch and consumption of large amounts of starch can increase glycemic index and load—2 determinants of T2D [[39], [40], [41]]. In the United States, potatoes are frequently consumed with fat or red/processed meats, which could also increase risk of T2D. Consumption of potatoes with high glycemic index could lead to lipotoxicity via activation of gluconeogenesis and increase free fatty acid production [42] with resulting reduction in insulin-stimulated glucose uptake and insulin resistance [43]. Fried potatoes could increase energy density and lead to obesity, another risk factor for T2D. However, potatoes are also rich in vitamin C and potassium, which can reduce blood pressure [2,3] and stroke risk [3,4].

### Strengths and limitations

Our study has several strengths, including a large sample size to detect smaller effect sizes; diversity in sex, race/ethnicity, education level, and geographic location of study subjects; the use of harmonized and single analytical plan for cohort-specific analyses; availability of major and relevant potential confounding factors; and the use of well-characterized and large United States cohorts for analyses. However, several limitations should also be noted. Most cohorts relied on self-reported diagnosis of T2D, and we cannot exclude misclassification of outcome in our data. We did not have separate data on consumption of baked, boiled, and mashed potatoes to evaluate their individual associations with T2D risk. Furthermore, we did not have data on the food composition of potato-containing meal for our analyses. In most cohorts, potato chips were combined with other forms of chips (i.e., tortilla); hence, we may have underestimated total potato consumption. Our analysis relied on a single baseline assessment of potato consumption and therefore could not account for change in dietary intakes over time. Although we adjusted for dietary factors including red/process meats, or overall dietary patterns using PDI, we cannot exclude the possibility that unmeasured and/or residual confounding could explain observed findings.

In conclusion, our data showed that consumption of total potato was not associated with T2D risk while a modest elevated risk of T2D was observed with fried potato consumption.

## Author contributions

The authors’ responsibilities were as follows – LD: designed research; LD, HDS, I-ML, JEB, RLM, JMG, LMS, JEM: conducted research; LD, XZ, JL, EK: analyzed data; and LD, HDS, I-ML, JEB, LMS, JEM: wrote the article; LD: had primary responsibility for final content; and all authors: read and approved the final manuscript.

## Conflict of interest

LD received investigator-initiated grant from the Alliance for Potato Research and Education (as principal investigator).

## Funding

This project was supported by a grant from the Alliance Potato Research and Education (principal investigator: LD). Parent studies were funded by the National Institute of Health and Mars Edge (COSMOS). The funding for participating cohorts were as follows: The Atherosclerosis Risk in Communities study has been funded in whole or in part with Federal funds from the National Heart, Lung, and Blood Institute, National Institutes of Health, and Department of Health and Human Services (75N92022D00001, 75N92022D00002, 75N92022D00003, 75N92022D00004, 75N92022D00005). The Coronary Artery Risk Development in Young Adults (CARDIA) study is supported by contracts HHSN268201800003I, HHSN268201800004I, HHSN268201800005I, HHSN268201800006I, and HHSN268201800007I from the National Heart, Lung, and Blood Institute. COSMOS is supported by an investigator-initiated grant from Mars Edge, a segment of Mars Incorporated dedicated to nutrition research and products, which included infrastructure support. COSMOS is also supported in part by grants AG050657, AG071611, EY025623, and HL157665 from the NIH and contracts 75N92021D00001, 75N92021D00002, 75N92021D00003, 75N92021D00004, and 75N92021D00005 through the Women’s Health Initiative at the NIH. MESA was supported by contracts 75N92020D00001, HHSN268201500003I, N01-HC-95159, 75N92020D00005, N01-HC-95160, 75N92020D00002, N01-HC-95161, 75N92020D00003, N01-HC-95162, 75N92020D00006, N01-HC-95163, 75N92020D00004, N01-HC-95164, 75N92020D00007, N01-HC-95165, N01-HC-95166, N01-HC-95167, N01-HC-95168, and N01-HC-95169 from the National Heart, Lung, and Blood Institute and by grants UL1-TR-000040, UL1-TR-001079, and UL1-TR-001420 from the National Center for Advancing Translational Sciences. A full list of participating MESA investigators and institutions can be found at http://www.mesa-nhlbi.org. This article has been reviewed and approved by the MESA Publications and Presentations Committee. PHS was funded by grants CA 097193, CA 34944, CA 40360, HL 26490, and HL 34595 from the National Institutes of Health (Bethesda, MD). WACS was funded by the National Heart, Lung, and Blood Institute. WHS is funded by NIH grants CA047988, CA182913, HL043851, HL080467, and HL099355.

## Data availability

CARDIA complies with data sharing requirements of the National Institutes of Health by providing limited-access data sets from various CARDIA examinations to the National Heart, Lung and Blood Institute BioLINCC. Data from CARDIA and ARIC are available through BioLINCC.
