# Supplementary material for: Potato Consumption and Risk of Type 2 Diabetes Mellitus: A Harmonized Analysis of 7 Prospective Cohorts
Source: J Nutr. 2024 Sep 16;154(10):3079–87. doi: 10.1016/j.tjnut.2024.07.020 (PMC12612587; doi:10.1016/j.tjnut.2024.07.020)
Supplement: Multimedia component 1 [file mmc1.pptx]

## Slide 1
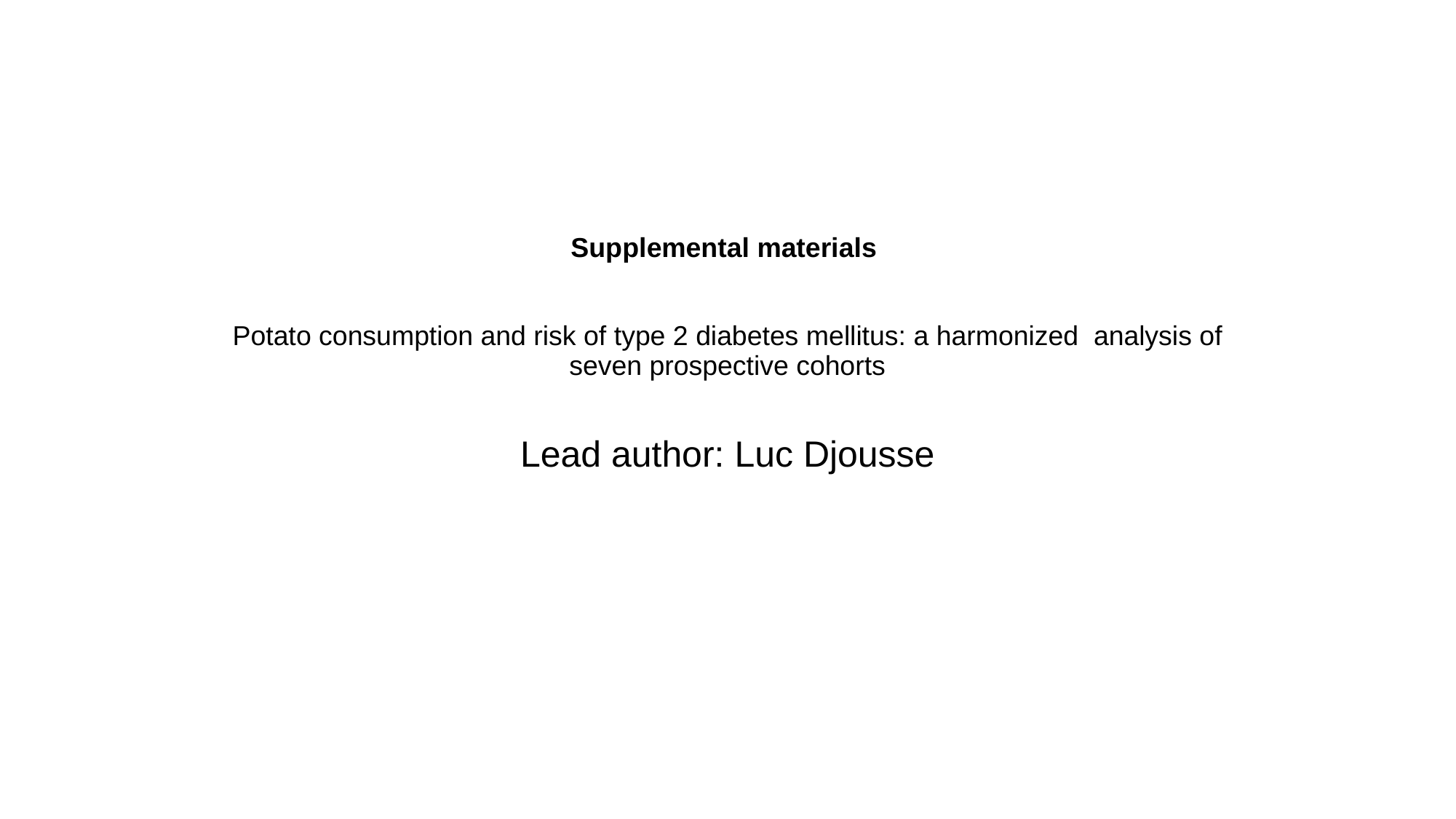

# Supplemental materials Potato consumption and risk of type 2 diabetes mellitus: a harmonized analysis of seven prospective cohorts
Lead author: Luc Djousse

## Slide 2
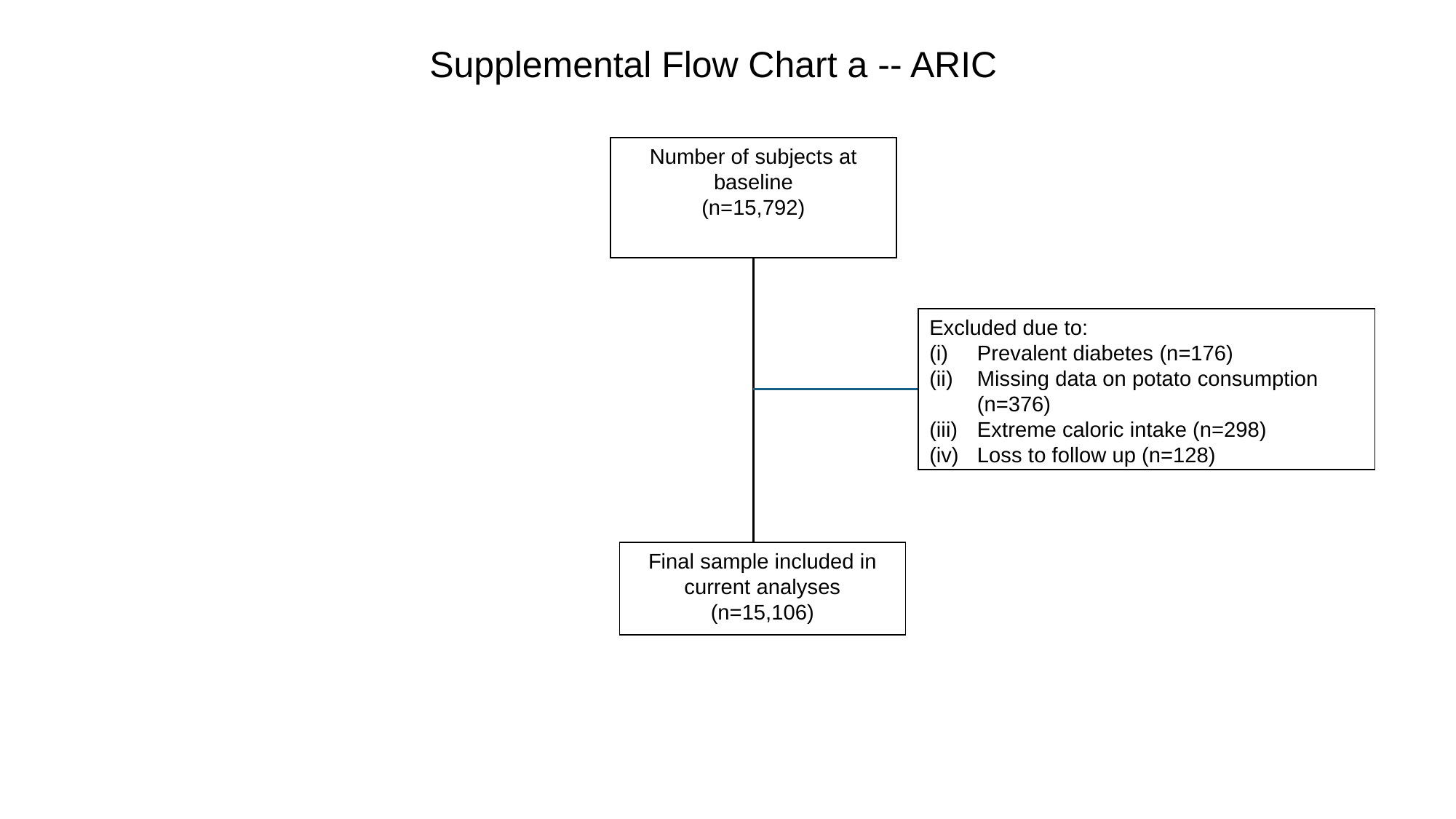

# Supplemental Flow Chart a -- ARIC
Number of subjects at baseline
(n=15,792)
Excluded due to:
Prevalent diabetes (n=176)
Missing data on potato consumption (n=376)
Extreme caloric intake (n=298)
Loss to follow up (n=128)
Final sample included in current analyses
(n=15,106)

## Slide 3
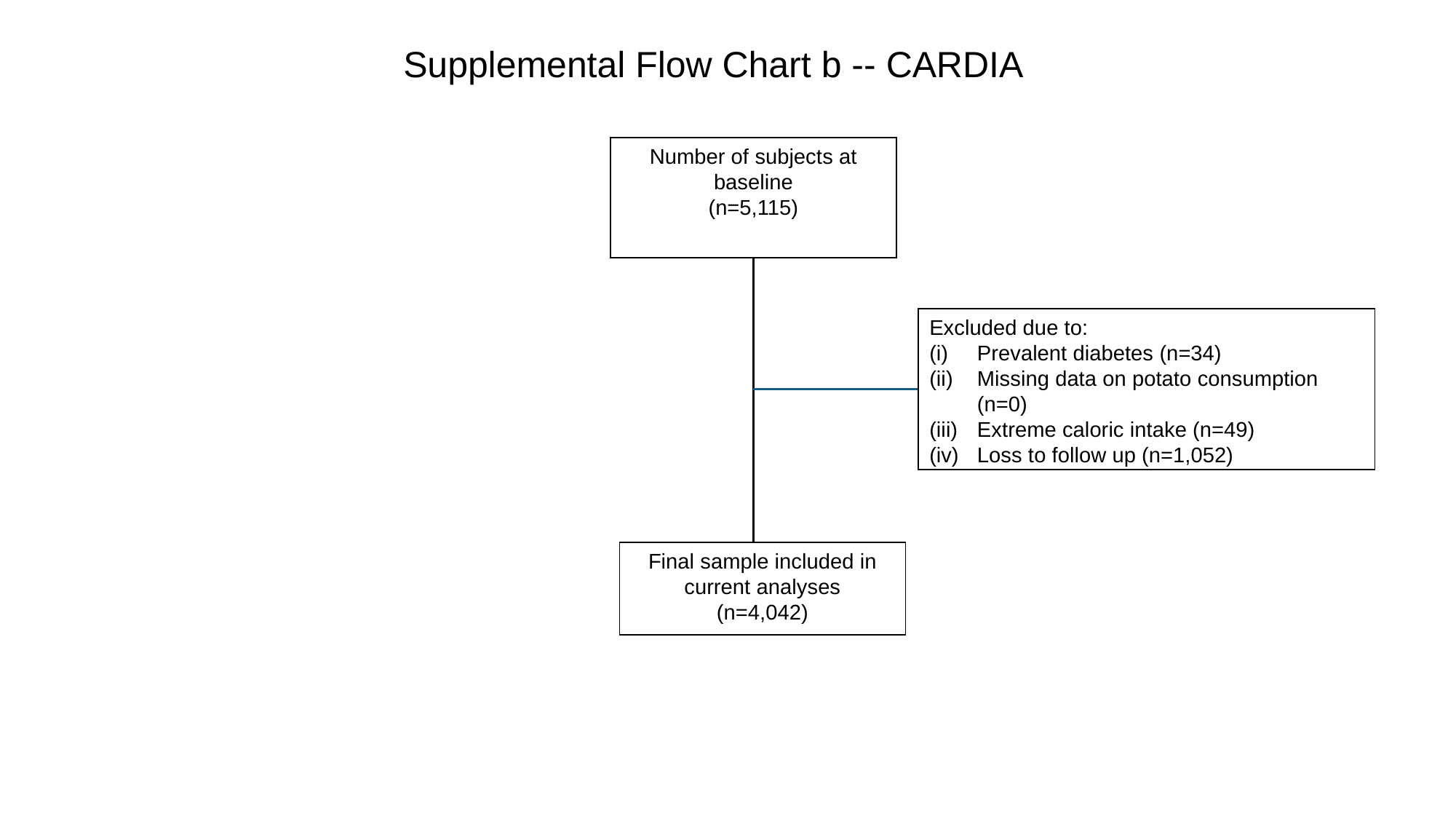

# Supplemental Flow Chart b -- CARDIA
Number of subjects at baseline
(n=5,115)
Excluded due to:
Prevalent diabetes (n=34)
Missing data on potato consumption (n=0)
Extreme caloric intake (n=49)
Loss to follow up (n=1,052)
Final sample included in current analyses
(n=4,042)

## Slide 4
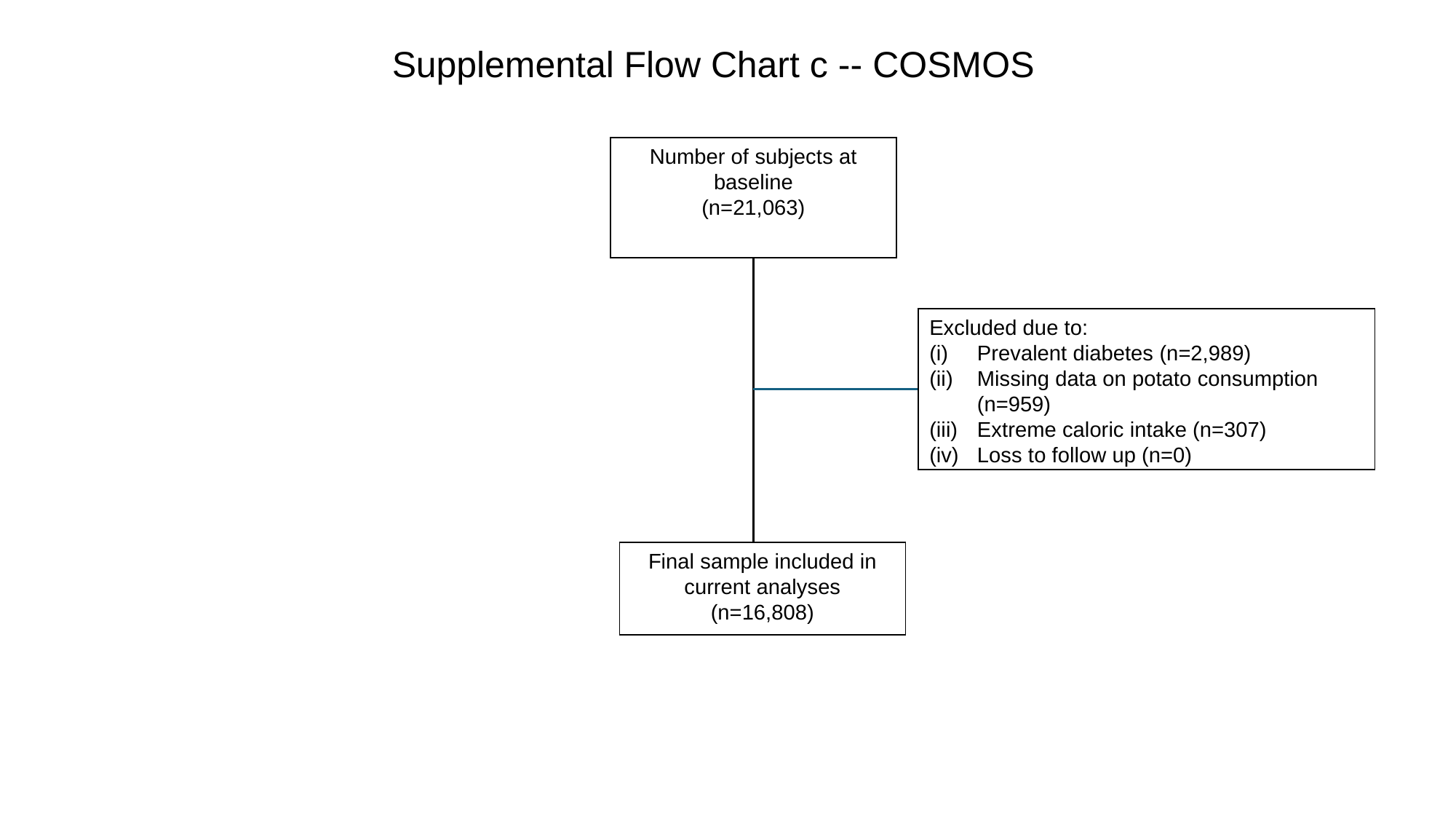

# Supplemental Flow Chart c -- COSMOS
Number of subjects at baseline
(n=21,063)
Excluded due to:
Prevalent diabetes (n=2,989)
Missing data on potato consumption (n=959)
Extreme caloric intake (n=307)
Loss to follow up (n=0)
Final sample included in current analyses
(n=16,808)

## Slide 5
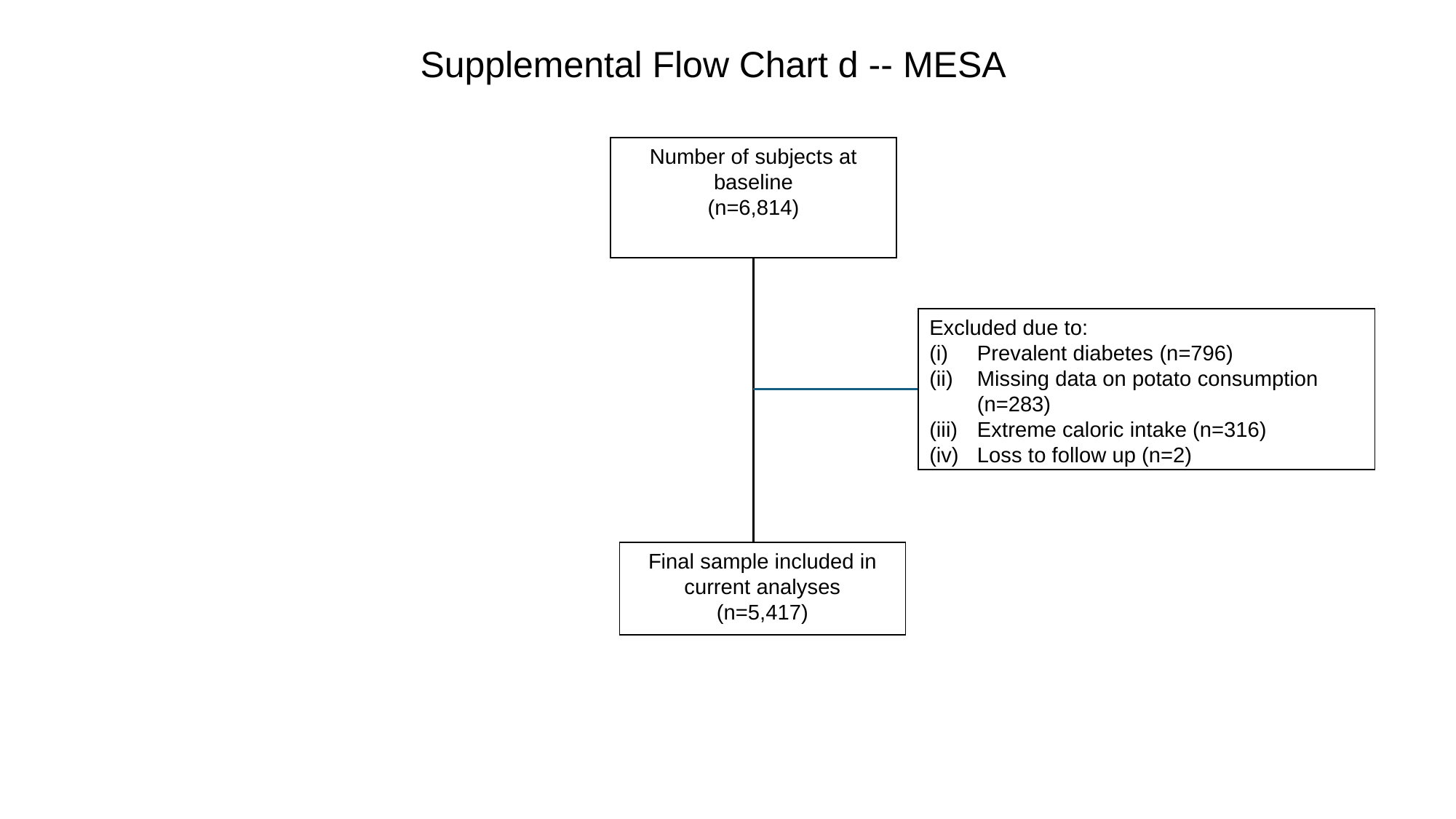

# Supplemental Flow Chart d -- MESA
Number of subjects at baseline
(n=6,814)
Excluded due to:
Prevalent diabetes (n=796)
Missing data on potato consumption (n=283)
Extreme caloric intake (n=316)
Loss to follow up (n=2)
Final sample included in current analyses
(n=5,417)

## Slide 6
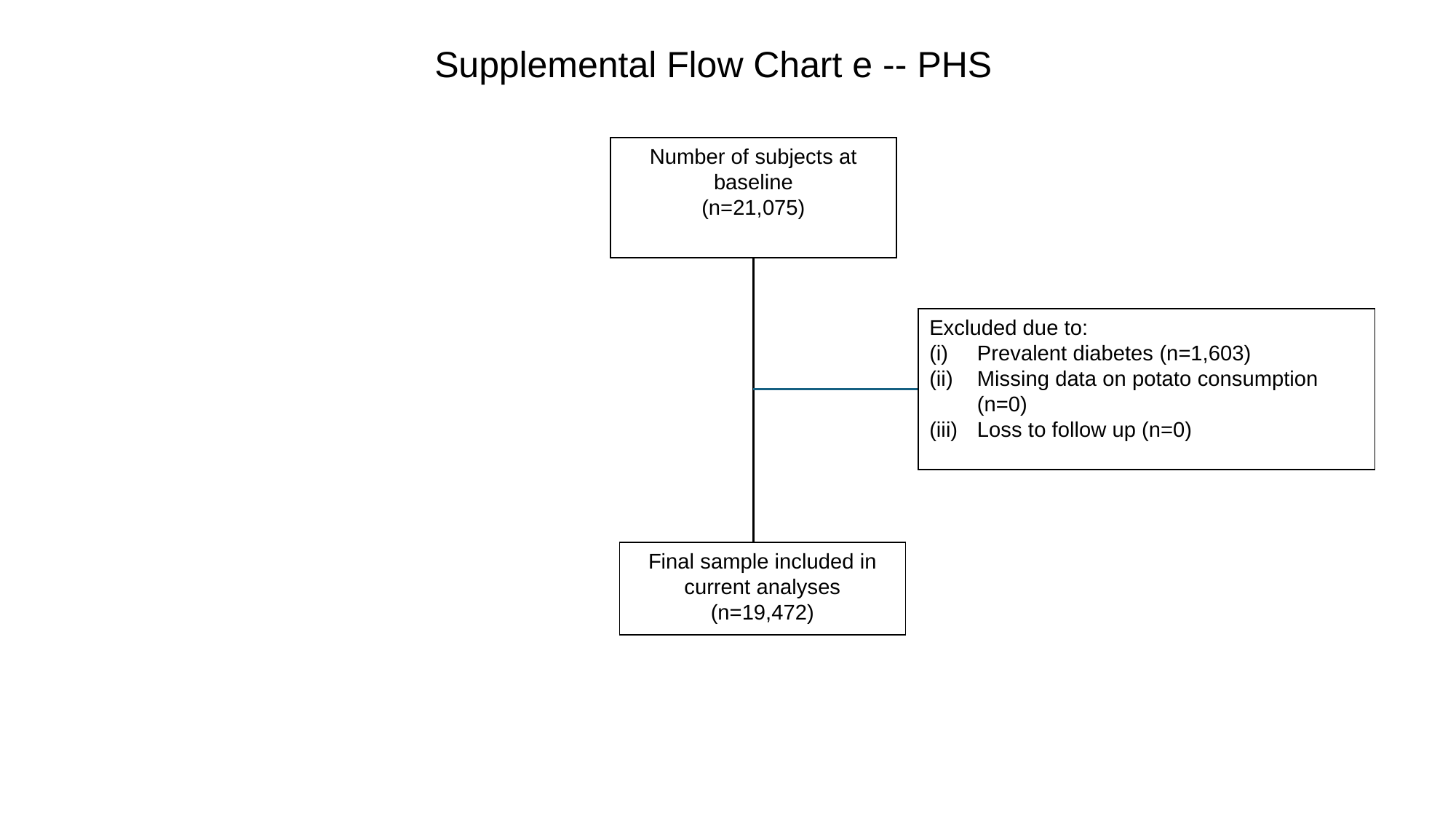

# Supplemental Flow Chart e -- PHS
Number of subjects at baseline
(n=21,075)
Excluded due to:
Prevalent diabetes (n=1,603)
Missing data on potato consumption (n=0)
Loss to follow up (n=0)
Final sample included in current analyses
(n=19,472)

## Slide 7
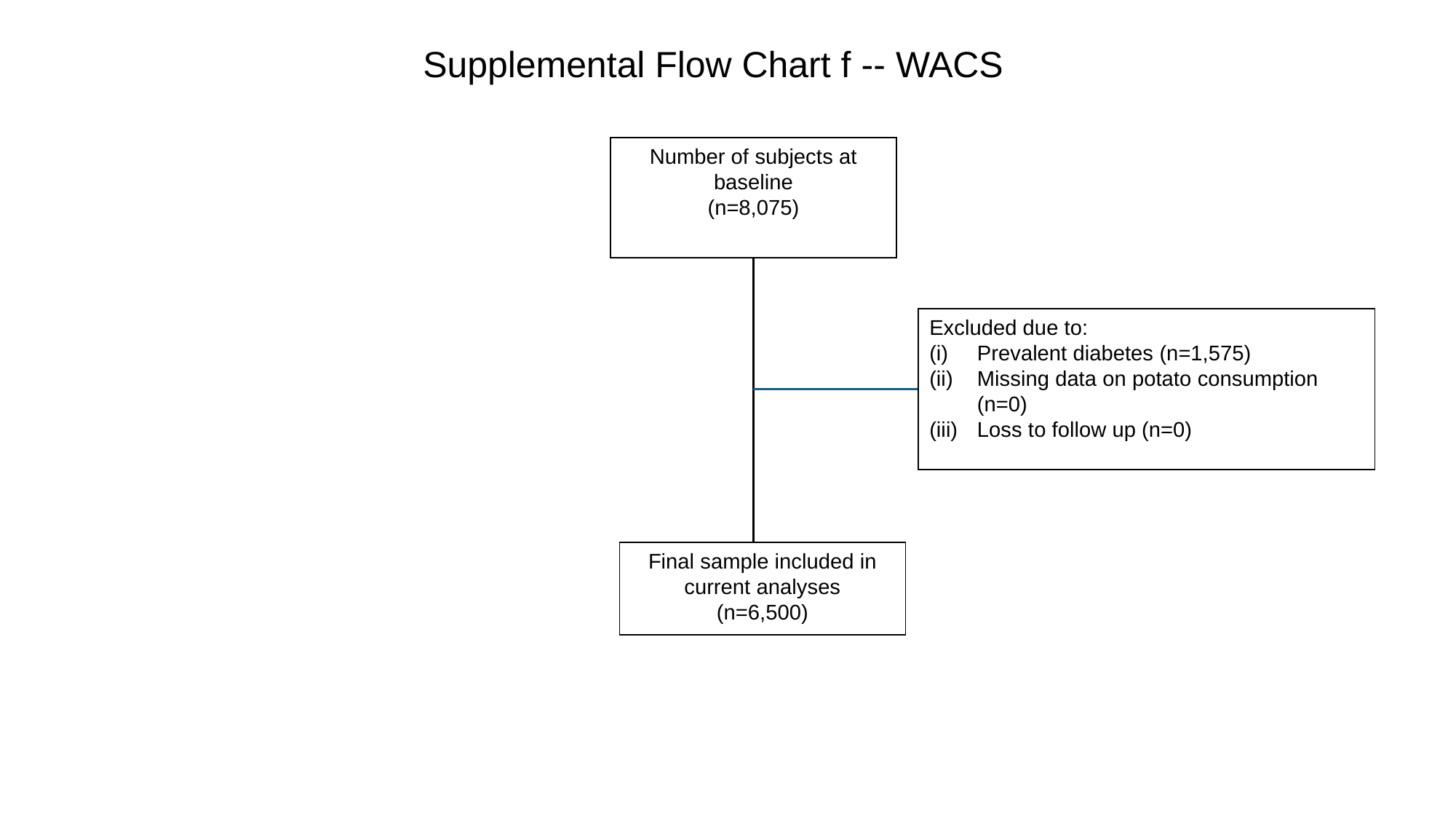

# Supplemental Flow Chart f -- WACS
Number of subjects at baseline
(n=8,075)
Excluded due to:
Prevalent diabetes (n=1,575)
Missing data on potato consumption (n=0)
Loss to follow up (n=0)
Final sample included in current analyses
(n=6,500)

## Slide 8
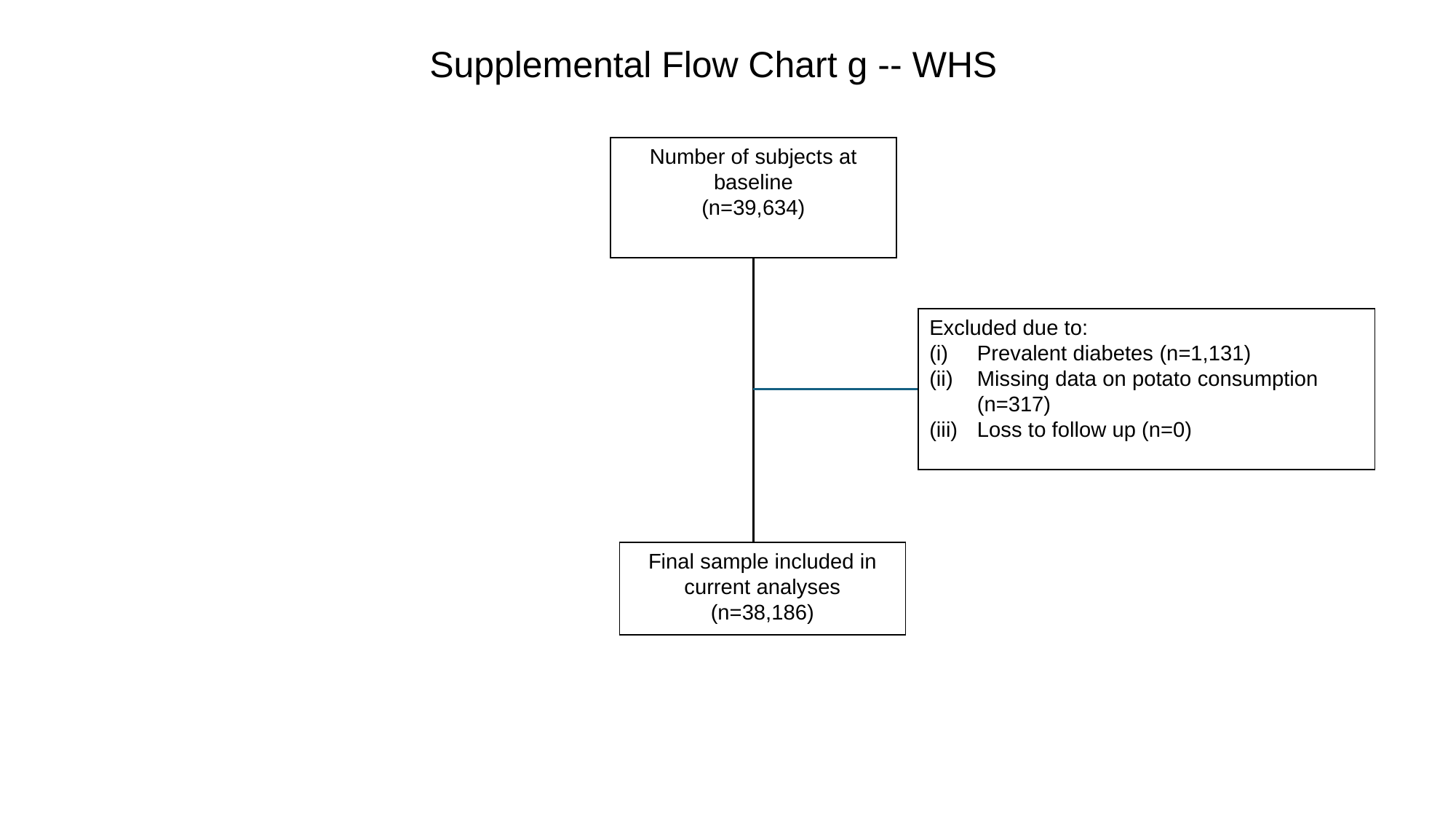

# Supplemental Flow Chart g -- WHS
Number of subjects at baseline
(n=39,634)
Excluded due to:
Prevalent diabetes (n=1,131)
Missing data on potato consumption (n=317)
Loss to follow up (n=0)
Final sample included in current analyses
(n=38,186)

## Slide 9
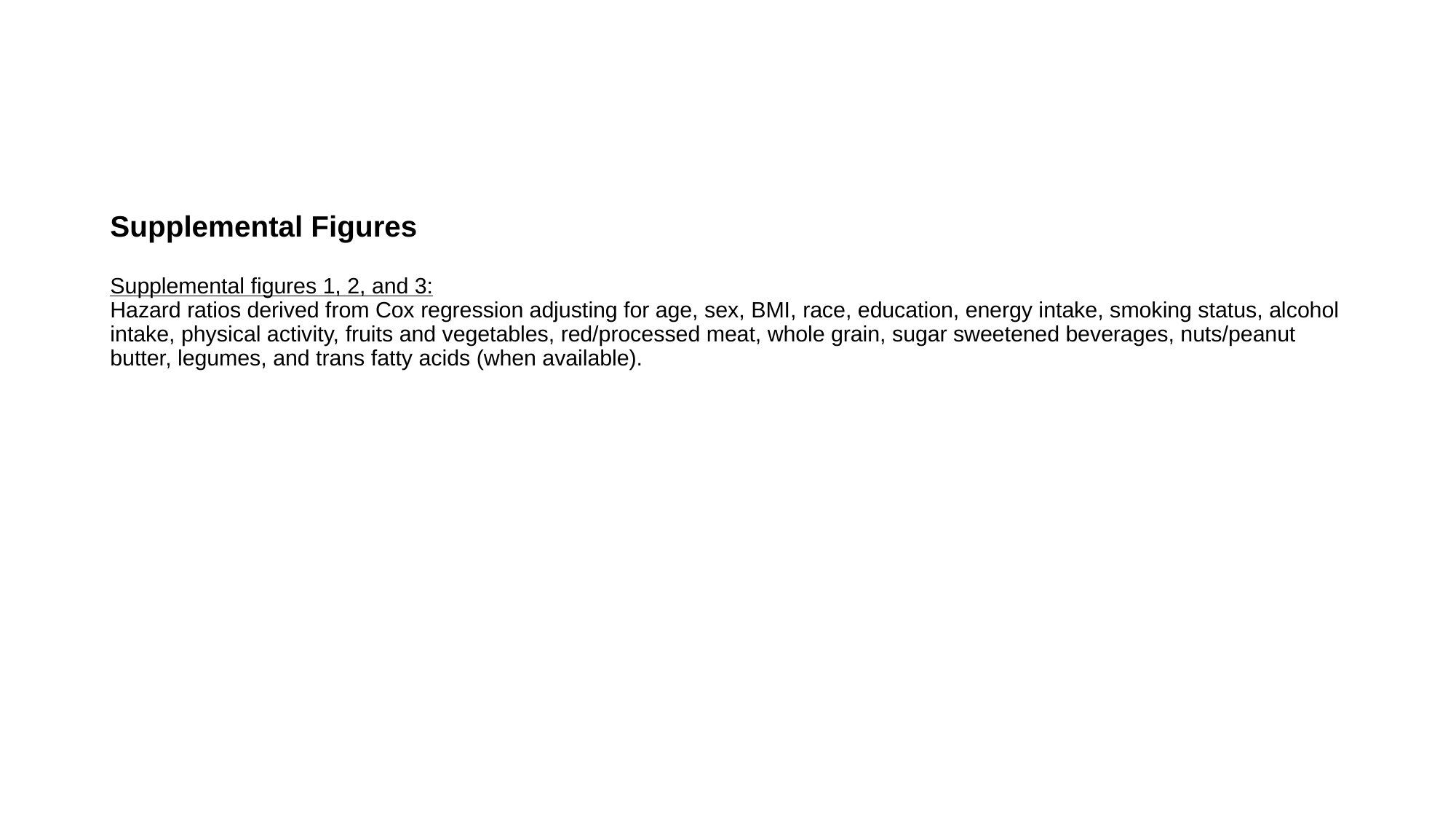

# Supplemental Figures Supplemental figures 1, 2, and 3: Hazard ratios derived from Cox regression adjusting for age, sex, BMI, race, education, energy intake, smoking status, alcohol intake, physical activity, fruits and vegetables, red/processed meat, whole grain, sugar sweetened beverages, nuts/peanut butter, legumes, and trans fatty acids (when available).

## Slide 10
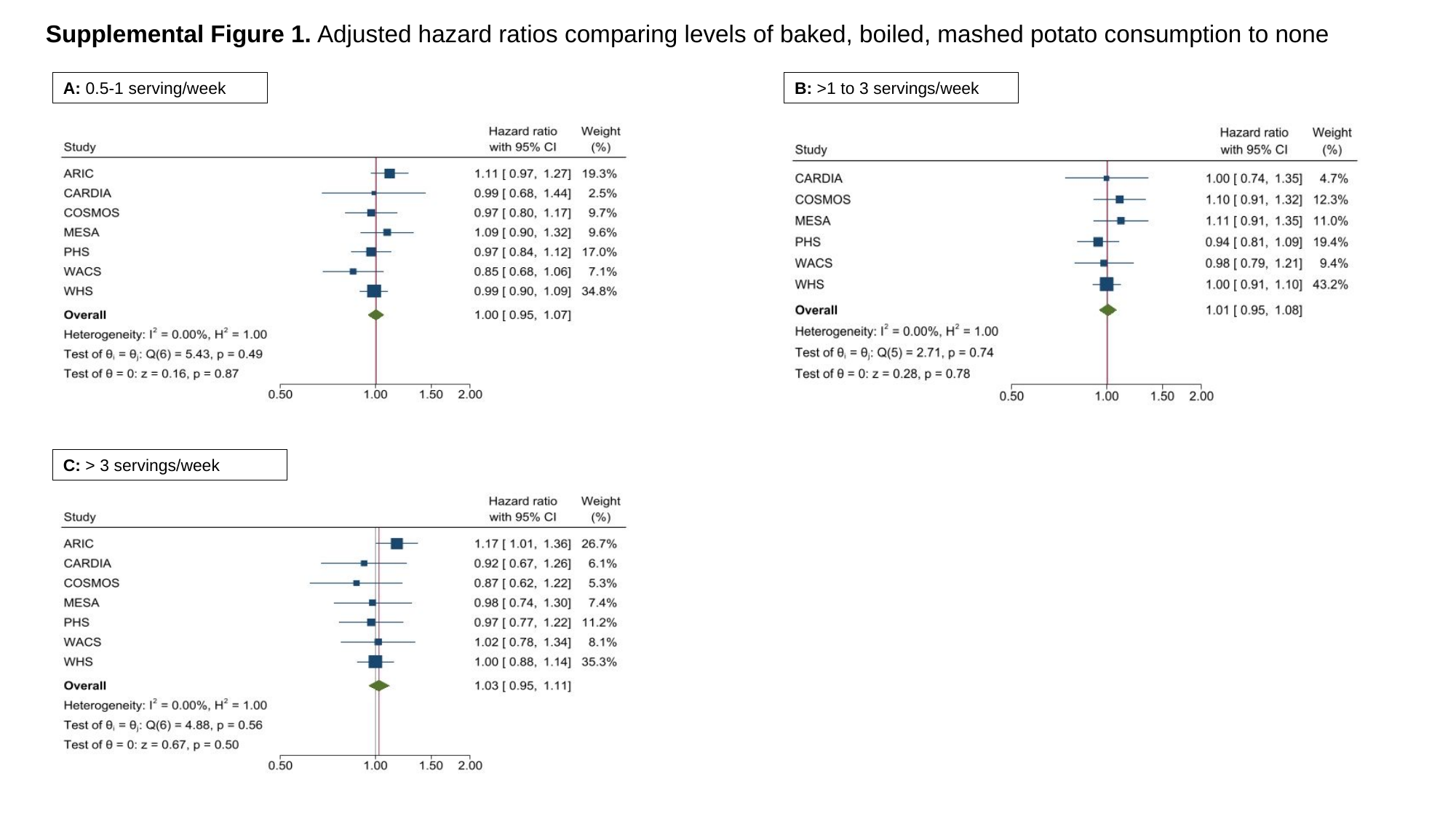

Supplemental Figure 1. Adjusted hazard ratios comparing levels of baked, boiled, mashed potato consumption to none
A: 0.5-1 serving/week
B: >1 to 3 servings/week
C: > 3 servings/week

## Slide 11
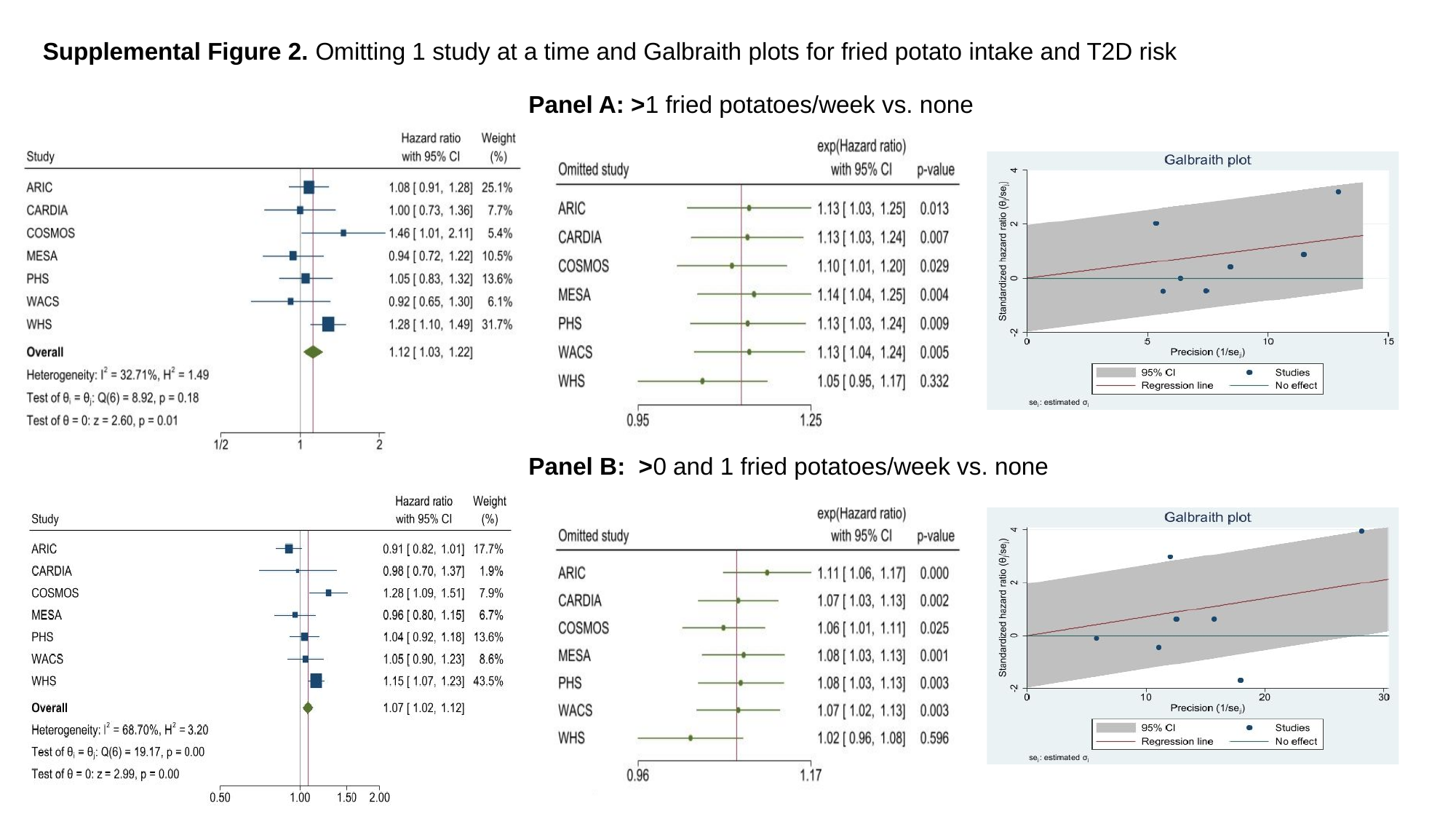

Supplemental Figure 2. Omitting 1 study at a time and Galbraith plots for fried potato intake and T2D risk
Panel A: >1 fried potatoes/week vs. none
Panel B: >0 and 1 fried potatoes/week vs. none

## Slide 12
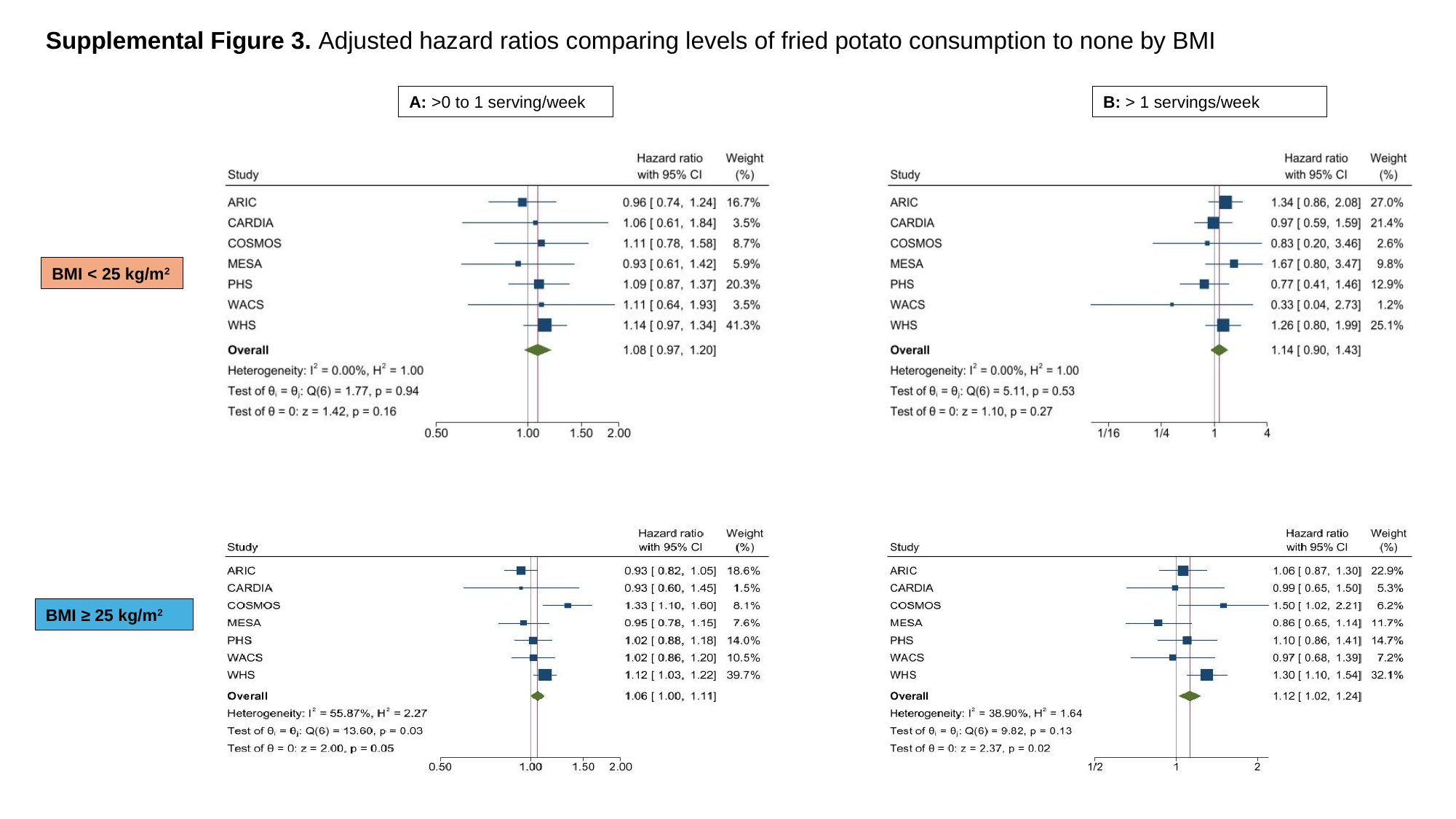

Supplemental Figure 3. Adjusted hazard ratios comparing levels of fried potato consumption to none by BMI
A: >0 to 1 serving/week
B: > 1 servings/week
BMI < 25 kg/m2
BMI ≥ 25 kg/m2
