# Supplementary material for: Potato Consumption and Risk of Type 2 Diabetes Mellitus: A Harmonized Analysis of 7 Prospective Cohorts
Source: J Nutr. 2024 Sep 16;154(10):3079–87. doi: 10.1016/j.tjnut.2024.07.020 (PMC12612587; doi:10.1016/j.tjnut.2024.07.020)
Supplement: Multimedia component 2 [file mmc2.docx]

| **Supplemental Table.** Characteristics of cohorts included in the analyses | | | | | |
| --- | --- | --- | --- | --- | --- |
| **Study Name** | **N (age range at enrollment)** | **Cohort baseline (y)** | **Assessment of potatoes** | **Assessment of T2D** | **Mean follow- up** |
| ARIC | 15,792 (45-64y) | 1987-1989 | Modified Willett semi-quantitative 66-item FFQ | Self-reported physician diagnosis, self-reported use of diabetes medications, a non-fasting blood glucose level ≥200 mg/dL, or a fasting blood glucose (FBG) ≥126 mg/dL | 8.1 years |
| CARDIA | 5,115 (18-30y) | 1985-1986 | Modified CARDIA diet questionnaire | Fasting glucose ≥7.0 mmol/l or the use of diabetes medications | 2.6 years |
| COSMOS | 21,442 (60+ y) | 2016-2018 | Willett FFQ |  | 3.6 years |
| MESA | 6,814 (45-84y) | 2000-2002 | modified Block-style 120-item FFQ. | Fasting glucose ≥126 mg/dL and/or reported use of diabetes medications | 13.8 years |
| PHS | 20,071 (40-85y) | 1999-2002 | Willett FFQ | Self-report of T2D diagnosis and/or use of diabetes medications | 10.7 years |
| WACS | 8,171 (40+y) | 1995-1996 | Willett FFQ | Self-report of T2D diagnosis and/or use of diabetes medications | 8.4 years |
| WHS | 39,876 (45+y) | 1993-1996 | Willett FFQ | Self-report of T2D diagnosis and/or use of diabetes medications | 21.4 years |
| ARIC: Atherosclerosis Risk in Communities  CARDIA: Coronary Artery Risk Development in Young Adults  COSMOS: COcoa Supplement and Multivitamin Outcomes Study  MESA: Multi-Ethnic Study of Atherosclerosis  PHS: Physicians’ Health Study  WACS: Women's Antioxidant Cardiovascular Study  WHS: Women’s Health Study | | | | | |
